# Kidney Transplantation in Older Recipients: One-Year Outcomes and Complications from a Single-Center Experience

**DOI:** 10.3390/jcm14186545

**Published:** 2025-09-17

**Authors:** Aleksandra Barbachowska-Kubik, Jolanta Gozdowska, Magdalena Durlik

**Affiliations:** Department of Transplantology, Immunology, Nephrology and Internal Medicine, Medical University of Warsaw, 02-091 Warsaw, Poland; ola.barbachowska@gmail.com (A.B.-K.);

**Keywords:** clinical complications, graft survival, kidney transplantation, older kidney recipients, patient survival, surgical complications

## Abstract

**Background/Objectives**: Each year, the number of kidney transplants (KT) performed in older recipients continues to rise. The process of aging may impact early post-transplant outcomes. The aim of this study was to analyze one-year outcomes, clinical and surgical complications, as well as patient and graft survival in senior recipients. **Methods**: This retrospective, observational study included a total of 270 participants who underwent KT during the period between January 2021 and April 2024. Recipients were divided into two groups: the older group (≥60 years; n = 75) and the younger group (<60 years; n = 195) and then analyzed during a one-year follow-up period. **Results**: Older recipients were characterized by a higher body mass index (MD = 1.77, CI95 [0.63; 2.91], *p* = 0.002), suffered more often from diabetes mellitus (RR = 2.94, CI95 [1.79; 4.82], *p* < 0.001), cardiovascular diseases (RR = 5.20, CI95 [2.90; 9.32], *p* < 0.001) and were more likely to receive a kidney from older (MD = 12.37, CI95 [8.94; 15.80], *p* < 0.001) deceased (*p* < 0.001) donors. Senior patients had more infections (*p* = 0.019) and surgical complications (RR = 1.81, CI95 [1.14; 2.87], *p* = 0.020), more cardiac events (RR = 2.28, CI95 [1.17; 4.43], *p* = 0.025), and a higher incidence of delayed graft function (*p* < 0.001) compared to younger patients. The estimated glomerular filtration rate (eGFR) was significantly lower in the older group both at initial hospital discharge (MD = −6.50, CI95 [−13.00; −3.00], *p* = 0.004) and at one-year follow-up (MD = −11.79, CI95 [−17.32; −6.25], *p* < 0.001). No differences were observed in the incidence of biopsy-proven acute rejection, cytomegalovirus replication, and polyomavirus replication. One-year patient and graft survival was 97.3% and 94.7% in the older group, and 98.5% and 96.9% in the younger group, respectively. **Conclusions**: Kidney transplantation in older recipients is safe in the short term. Although eGFR was lower in the older group, it remained within an acceptable range.

## 1. Introduction

Kidney transplantation (KT) remains the most effective treatment of end-stage kidney disease (ESKD) [1]. Moreover, compared to patients on dialysis, kidney transplant recipients have a longer expected lifespan across all age groups [2].

Although the percentage of kidney transplants is still the highest among younger patients (15% in the 20–44-year age group vs. 4% in the 65–74-year age group), each year the number of kidney transplants in older recipients is growing [2,3]. One of the reasons for that is the aging of the population. According to the World Health Organization (WHO), by 2050, 22% of the world’s population will be over 60 years of age [4]. The aging process affects most organs and systems. Additionally, older individuals often present with multiple comorbidities—such as diabetes mellitus (DM), cardiovascular diseases (CVD), and frailty- all of which may reflect on early post-kidney transplant surgical and clinical complications, as well as patient and graft-survival. Therefore, the older kidney transplant recipients may require a distinct clinical approach that takes these variables into account. Previous studies present inconclusive findings, especially regarding surgical complications and biopsy-proven acute rejection. Moreover, several aspects (for instance, cardiac complications, presence of cytomegalovirus replication, or polyomavirus replication) need further investigation. This single-center study aimed to analyze early (one-year) post-transplant outcomes, clinical and surgical complications, as well as patient and graft survival in senior patients.

## 2. Materials and Methods

### 2.1. Study Population

**Figure 1:**
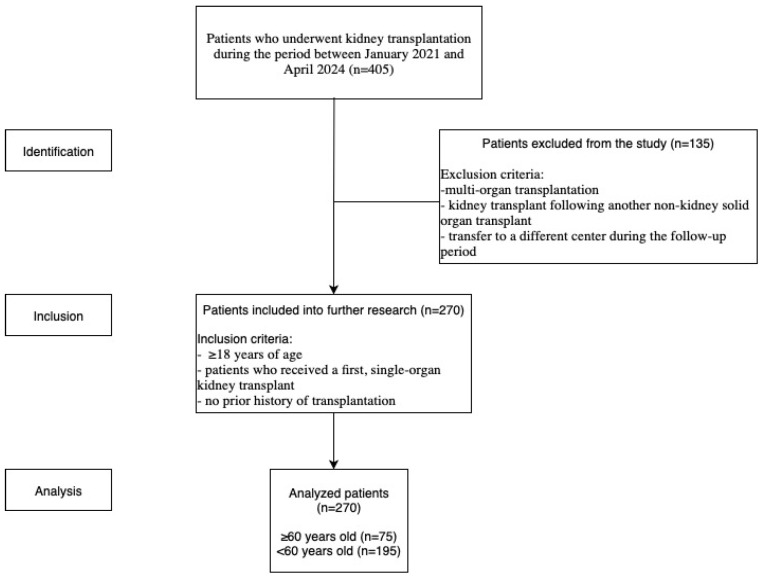
STROBE Flow Chart of Patient Inclusion and Exclusion Criteria during 12-month observational period.

In this retrospective observational study, we analyzed patients who underwent kidney transplantation at Infant Jesus Clinical Hospital, Warsaw, Poland, during the period between January 2021 and April 2024. A total of 405 kidney transplant procedures (KTs) were performed. Patients who were ≥18 years of age received a first, single-organ kidney transplant, and had no prior history of transplantation were included in further research. The exclusion criteria included multi-organ transplantation, KT following another non-kidney solid organ transplant, and transfer to a different center during the follow-up period, which resulted from the nationwide mandatory organ allocation framework for kidney transplantation. The study included 270 patients. Participants were divided into two groups according to age (older recipients ≥60 years, n = 75; younger recipients <60 years, n = 195) and then compared. All information was accessed through medical records and laboratory test results. The selection process of the study cohort is outlined in the STROBE flow diagram (Figure 1).

Our data included demographics, cause of ESKD, type of dialysis (if applicable), duration of dialysis, comorbidities at the time of transplantation, and type of immunosuppressive regimens. Donor-related data such as age, sex, body mass index (BMI), comorbidities (diabetes mellitus, hypertension, cardiovascular diseases), donor type (living or deceased), as well as panel reactive antibody (PRA), human leukocyte antigen (HLA) mismatches, and Remuzzi score in zero-time kidney biopsy were also considered in the analysis.

Outcome variables included surgical complications, clinical complications such as newly diagnosed post-transplant diabetes mellitus, biopsy-proven acute rejection (BPAR), infection that occurred during the initial hospitalization, infections which required hospitalization during the follow-up period, cardiac events, cytomegalovirus replication, and polyomavirus replication, length of initial hospitalization, number of hospitalizations during the follow-up period, patient and graft survival and post-transplant graft function.

The follow-up period was 12 months. The study was conducted in full accordance with the principles of the Declaration of Helsinki and the Declaration of Istanbul.

### 2.2. Definitions

Older patients were defined as those aged ≥60 years, based on the United Nations definition [5].

An expanded criteria donor was defined as a deceased donor aged over 60 years, or a donor aged over 50 years with at least two of the following: hypertension, serum creatinine ≥ 0.133 mmol/L, or death due to stroke. The Remuzzi score was calculated by the addition of four different parameters: glomerular global sclerosis (0–3), tubular atrophy (0–3), interstitial fibrosis (0–3), arterial and arteriolar narrowing (0–3). It was included in the analysis to assess graft quality at the time of transplantation, as well as to evaluate the extent of chronic histological injury, which could influence graft function. Infection was diagnosed based on positive cultures and laboratory findings, caused by bacterial, viral, or fungal pathogens. Throughout the follow-up period, during both the initial hospitalization and any subsequent infection-related hospitalizations, the site of infection (e.g., urinary tract, wound, pneumonia, or sepsis of unknown origin) and the specific pathogen (bacteria, virus, fungus, or unidentified) were identified. Polyomavirus replication was determined in serum samples. Both polyomavirus replication and cytomegalovirus replication were monitored in the third month post-transplant, and subsequently every 3 to 6 months or in the presence of symptoms suggestive of viremia. The diagnosis of BPAR was made based on histological findings obtained during the protocol biopsy at 3 months post-transplant or when acute rejection was clinically suspected. Graft function was assessed based on the: primary non-function (PNF), presence of delayed graft function (DGF) and immediate graft function (IGF), as well as serum creatinine (sCr) and estimated glomerular filtration rate (eGFR), both measured at the end of initial hospitalization and after 12 months. Surgical complications, defined as postoperative events directly related to the surgical procedure that required surgical intervention, were divided into 3 subgroups: vascular (which included renal graft vessel thrombosis, renal graft vessel stenosis; iliac artery dissection, iliac artery thrombosis and iliac artery pseudoaneurysm), urological (urinal leakage, ureteral obstruction, lymphocele) and those related to surgical wound healing (wound dehiscence, wound infection, wound prolonged healing). Delayed graft function (DGF) was defined as the need for dialysis within the first week after KT. Immediate graft function (IGF) was defined as a functioning allograft immediately post-transplant, characterized by the appearance of diuresis, a progressive decrease in serum creatinine and no requirement for dialysis within the first 7 days. Primary non- function (PNF) was defined as the complete absence of graft function following transplantation, with the patient remaining dialysis-dependent. Post-transplant diabetes mellitus (PTDM) was diagnosed based on the 2013 International Consensus Meeting on Post-transplant Diabetes Mellitus and included fasting glucose > 7 mmol/L on more than one occasion or random glucose > 11.1 mmol/L with symptoms or 2-h glucose after a 75-g OGTT of >11.1 mmol/L or hemoglobin A1c (HbA1c) ≥ 6.5% [6]. Cardiac events, defined as clinically significant cardiovascular complications occurring within 12 months after transplantation, included myocardial infarction, ischemic heart disease, new-onset congestive heart failure, and arrhythmia. The standard immunosuppressive regimen consisted of a calcineurin inhibitor (tacrolimus or cyclosporine), mycophenolate mofetil (MMF), and corticosteroids. The choice of induction therapy depended on the patient’s immunological risk and comorbidities: no induction was used in low-risk recipients, basiliximab in those with intermediate risk, and thymoglobulin (ATG) in high-risk recipients. Immunosuppressive treatment was not modified based on recipient age.

### 2.3. Statistical Analysis

Continuous variables were summarized as mean ± standard deviation (SD) or median and interquartile range (IQR), depending on normality. Categorical variables were presented as n (%). Normality was evaluated with the Shapiro-Wilk test, along with assessments of skewness and kurtosis. Levene’s test was applied to assess homogeneity of variances. Comparisons between age groups were made with Student’s *t*-test, Welch *t*-test, Mann-Whitney U test, Pearson’s chi-square test or Fisher’s exact test, as appropriate. Mean/median difference (MD) was used to measure the difference between age groups in case of continuous variables and relative risk (RR) was used to measure the difference in case of proportions, both MD and RR were presented with 95% confidence intervals (CI). Alpha of 0.05 was used for statistical significance. All analyses were performed using R software (R4.4.2).

## 3. Results

### 3.1. Demographics

Of the 405 KT procedures, 270 patients met the inclusion criteria and were included in the final analysis. Recipients were divided into two groups based on their age: the older group (≥60 years) and the younger group (<60 years). The older group consisted of 75 patients (28%; 29 females (38.7%) and 46 males (61.3%)) with the mean age of 65 years, while the younger group was represented by 195 patients (72%; 80 females (41%) and 115 males (59%)), with a mean age of 41 years. Recipients in the older group had a significantly higher body mass index (BMI) (MD = 1.77, CI95 [0.63; 2.91], *p* = 0.002), more often suffered from diabetes mellitus (DM) (34.7% vs. 11.8%, RR = 2.94, CI95 [1.79; 4.82], *p* < 0.001) and cardiovascular diseases (37.3% vs. 7.2%, RR = 5.20, CI95 [2.90; 9.32], *p* < 0.001). The etiology of end-stage kidney disease (ESKD) differed between the groups, *p* < 0.001. Diabetes mellitus (DM), autosomal dominant polycystic kidney disease (ADPKD), hypertension (HT) and undetermined causes were more frequent among patients ≥60 years compared to patients <60 years (DM: 17.3% vs. 9.2%, ADPKD: 16.0% vs. 13.3%, HT: 8.0% vs. 4.1%, undetermined:25.3 vs. 7.7%). Chronic glomerulonephritis was the main cause of ESKD among patients <60 years.

All patients received a standard immunosuppression protocol consisting of steroids, calcineurin inhibitors (CNI), and mycophenolate acid. Induction immunosuppression was used more frequently in younger recipients compared to the older ones (*p* = 0.014). Sixty-one patients (22.5%) received thymoglobulin (ATG) induction therapy, 10.6% from the older group (8 recipients) and 27.1% from the younger group (53 recipients). Thirty patients (11%) received basiliximab prior to transplantation, 12% from the older group (9 recipients) and 10.7% from the younger group (21 patients).

Both groups received kidney transplants predominantly from deceased donors (DDKT), (group ≥60 years: 94.7%, group <60 years: 72.3%). The mean donor age was significantly higher in the older group (58 years vs. 45 years, MD = 12.37, CI95 [8.94; 15.80], *p* < 0.001). Additionally, expanded criteria for DDKD were four times more common in the group ≥60 years (54.9% vs. 13.5%, RR = 4.08, CI95 [2.55; 6.51], *p* < 0.001).

No statistically significant difference was found in Panel Reactive Antibody (PRA) and number of Human Leukocyte Antigens (HLA) mismatches.

The demographic and baseline characteristics of studied groups are presented in Table 1.

### 3.2. Clinical Complications

Table 2 presents a comparison of post-transplant outcomes and complications between the two groups. The Remuzzi score was higher by 1.00 among patients aged ≥60 years (MD = 1.00, CI95 [0.00; 1.00], *p* < 0.001). The mean Remuzzi score was 2.53 in the older group and 1.47 in the younger group. The first hospitalization was longer by 5 days on average among patients ≥60 years (MD = 5.00, CI95 [2.00; 6.00], *p* < 0.001). The older group had a higher infection rate in the early postoperative period compared to the younger group; however, the *p*-value of 0.057 does not meet the standard criterion for statistical significance (*p* < 0.05), though it suggests a marginal or borderline effect. The most common site of infection in both groups was the urinary tract. Patients aged ≥60 years had more hospitalizations within 12 months compared to patients <60 years (MD = 1.00, CI95 [0.00; 1.00], *p* = 0.002). Moreover, the proportion of patients hospitalized due to infection was 65% higher among patients ≥60 years compared to patients < 60 years (34.7% vs. 21.0%, RR = 1.65, CI95 [1.09; 2.49], *p* = 0.030). Bacteria were the most common cause of infection, and the urinary tract was the main site of the infection in both groups. A significant difference was confirmed for PTDM occurrence, *p* < 0.001. PTDM was more common in the group ≥60 years compared to the group <60 years (PTDM: 29.3% vs. 20.5%). Cardiac events were twice as common among patients ≥60 years compared to patients <60 years (18.7% vs. 8.2%, RR = 2.28, CI95 [1.17; 4.43], *p* = 0.025). Arrhythmia was the most common cardiac complication in both groups. No statistically significant differences were found in terms of cytomegalovirus (CMV) and polyomavirus (BKV) replication, as well as biopsy proven acute rejection (BPAR).

### 3.3. Surgical Complications

The proportion of surgical complications was higher among the older group (30.7% vs. 16.9%, RR = 1.81, CI95 [1.14; 2.87], *p* = 0.020). The two main complications were vascular (14.7% in the older group vs. 6.7% in the younger group) and urological (13.3% in the older group vs. 7.7% in the younger group).

### 3.4. Graft Function

**Figure 2:**
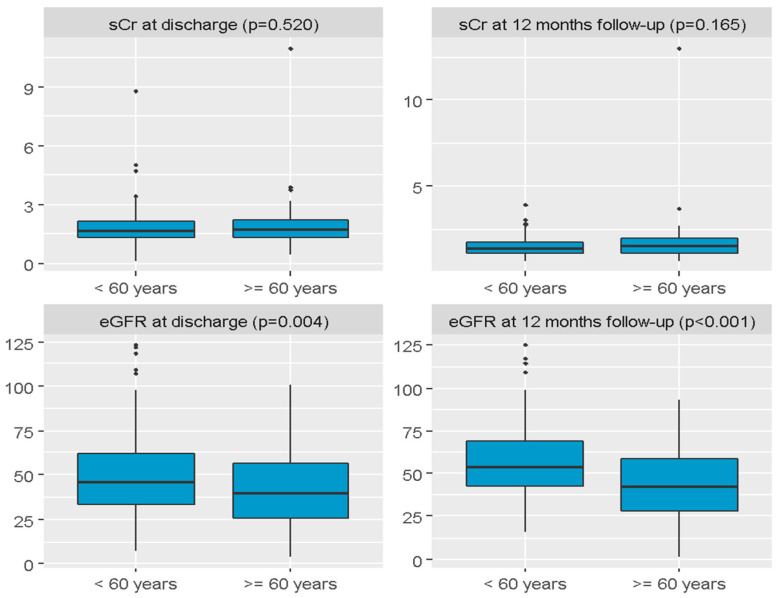
Boxplot charts presenting distribution of serum creatinine and estimated glomerular filtration rate at both discharge and 12 months after the transplantation in split to patients < 60 years and patients ≥ 60 years. sCr—serum creatinine; eGFR—estimated glomerular filtration rate (mL/min/1.73 m^2^).

A significant difference between groups was confirmed for graft function, *p* < 0.001. Primary non-function (PNF) was observed in three patients aged ≥60 years and two patients aged <60 years. Immediate graft function was less frequent in the ≥60 group compared to <60 group (38.7% vs. 65.6%), consequently delayed graft function was more frequent in the ≥60 group (57.3% vs. 33.3%). There was no statistical difference in the duration of DGF. The estimated glomerular filtration rate (eGFR) at hospital discharge after KT was lower among patients ≥60 years compared to patients <60 years (MD = −6.50, CI95 [−13.00; −3.00], *p* = 0.004). Similarly, eGFR at 12 months follow-up was lower in the older group (MD = −11.79, CI95 [−17.32; −6.25], *p* < 0.001). Figure 2 presents the distribution of serum creatinine (sCr) and eGFR at both discharge and 12 months post-transplantation, split by age group (<60 years and ≥60 years).

### 3.5. Survival Analysis

One-year patient and graft survival rates were comparable between the two groups (one-year patient survival: RR 0.99, 95% CI: [0.95–1.03]; *p* = 0.62; one-year graft survival: RR 0.98, 92% CI: [0.92–1.03]; *p* = 0.472). One-year patient survival was 97.3% in the older group and 98.5% in the younger group. One-year graft survival in patients ≥60 years of age was 94.7% and 96.9% in patients <60 years.

## 4. Discussion

This single-center retrospective study aimed to evaluate one-year outcomes, as well as clinical and surgical complications, in older kidney transplant recipients in comparison to younger patients. Numerous differences were found between the groups; however, our main findings indicate that kidney transplantation in older recipients is relatively safe and beneficial in the short-term perspective.

Among baseline characteristics of the patients, donor age was significantly higher in older recipients compared to younger ones, in both LDKT and DDKT groups. Expanded-criteria donors were also more often allocated to patients ≥60 years of age. Moreover, it was reflected in the Remuzzi score which was higher by 1.00 point compared to patients <60 years of age. This presents the tendency to allocate marginal donors, such as older individuals, to older KT recipients [7,8].

Since age is a well-known risk factor not only for type 2 diabetes mellitus in the general population, but also for post-transplant diabetes mellitus, it is not surprising that this correlation was also found in our study. PTDM was present in 29.3% patients aged ≥60 years, and in 20.5% of patients aged <60 years (*p* < 0.001), which corresponds with the outcomes of other studies [9,10].

Infectious complications are particularly important, as they are one of the main causes of death with a functioning graft in older kidney transplant recipients [11,12]. Different percentages of infections in older recipients have been reported, with some reaching as high as 92.3% within the first year after kidney transplantation [13]. In our study, infection in the early postoperative period did not meet the threshold for statistical significance; however, the number of hospitalizations due to infection during the follow-up period was significantly higher among older patients. Similar to Kim et al., the most common causative pathogens were bacteria, and the most frequent site was the urinary tract [14].

Cytomegalovirus replication/disease has been associated with lower death-censored graft survival in the first year after KT [15]. In a large, retrospective study conducted by Deina et al., age was identified as one of the risk factors for CMV infection [15]. Jankowska et al. also observed a trend toward more CMV infections in the older population [9]. However, different studies did not reach the same conclusion [16,17]. In our study, cytomegalovirus (CMV) infection did not differ between older and younger recipients, although it might be due to the relatively small group of participants and a short follow-up period.

Another viral infection which remains a challenging aspect after the kidney transplantation is BK polyomavirus (BKV) nephropathy. Although it has been associated with older recipient age, the level of evidence remains low [18]. In this study, no correlation between age and BKV replication was proven. Additionally, there are not many studies addressing the subject of BKV nephropathy in older kidney transplant recipients, thus more research is needed in this field.

Various studies have been inconclusive in terms of biopsy-proven acute rejection (BPAR). Doucet et al. found BPAR less frequent in older patients, which could be expected, when taking into consideration age-related changes in the immune system [19]. In contrast, some studies did not observe a significant difference in PBAR occurrence [11,16,20]. In our study, BPAR was also similar between both groups (22.1% of younger patients and 20% of older patients). It is plausible that the use of induction immunosuppression, particularly thymoglobulin, which was more frequently administered to younger patients (27.1% vs. 10.7%), contributed to the observed outcomes. Additionally, over time, more differences in terms of the immunosuppression approach might be observed, with a tendency to reduce calcineurin inhibitors in older patients. Thus, there is a need for more research in this regard.

In this study, cardiac events in the post-transplantation period were twice as common among patients ≥60 years compared to patients <60 years. Interestingly, in both groups, newly diagnosed arrhythmia was the main cardiac complication. In research conducted by Gozdowska et al. [21], living donor kidney transplant recipients were at lower risk of cardiovascular complications, compared to deceased donor kidney transplant recipients, of which the highest risk was during the first year. Not surprisingly, age, male gender, and frequent smokers were associated with a higher risk of such events [21]. It is common knowledge that PTDM, as well as type 2 diabetes mellitus, often followed by micro- and macroangiopathy conditions more often observed in the older group- could be additional important factors explaining the observed outcomes. Since cardiovascular diseases remain one of the leading causes of death in older kidney transplant recipients [11,12,20,22], there should be more careful pretransplant evaluation, focusing on cardiovascular diseases.

Similarly to other studies [11,23], delayed graft function (DGF) was more frequently observed in the group ≥60 years; however, there was no difference in terms of duration of DGF. Kidney graft function, measured with the eGFR formula, was poorer in older recipients at the end of first hospitalization, as well as after 12-month follow-up. However, in both groups, eGFR increased during that time. In our opinion, eGFR of 45 mL/min/1.73 m^2^ at the end of the first year post-KT is still an acceptable outcome for older recipients.

Another important aspect which has been a major concern in terms of KT in senior patients, is surgical complications. Many studies, including ours, have indeed observed an increased number of complications in older recipients [11,24]. Hernandez et al., in a study conducted on 870 cadaveric kidney transplants, reported that older recipients were more prone to urinary leak [25], which corresponds to our study results, where patients ≥60 years of age tended to have urologic complications. The reason for this remains unclear; however, it might be connected with elevated BMI and longer time on dialysis, both of which are more often present in senior patients [24,25]. In addition, it is possible that a higher number of surgical complications has influenced the initial hospital stay, which was on average 5 days longer in senior patients.

In our study, one-year patient survival and one-year graft survival were comparable between both groups. Therefore, in the short-term observation KT in senior recipients might be considered both beneficial and safe. Furthermore, Silva et al., in a retrospective study, did not find a correlation between age and one-year mortality [22]. Naturally, over time, patient survival rates tend to favor young recipients due to their longer life expectancy [9,11,19], and survival rates among older recipients decrease. However, in a study conducted by Jankowska et al., death-censored graft survival did not differ between the groups [9].

Our findings highlight key differences between younger and older recipients, particularly in terms of surgical complications, infection rates, PTDM occurrence and cardiac events. All of them were more likely presented in older recipients, and had an impact on graft function, which highlighted the need for careful monitoring of this group, particularly in the early post-transplant period.

The study has a number of limitations. Firstly, it is a single-center study with a relatively small group of participants (75 older and 195 younger KT recipients), which limits the extrapolation of the results to other populations. Furthermore, the disproportion in group sizes (the smaller number of senior patients compared to younger ones), as well as in donor characteristics (markedly fewer LDKT and more DDKT among older recipients) limits the strength of comparisons between the groups. Subsequently, the retrospective nature of the research influences the reliability of available data, or the lack of them, which limits the scope of the results. Another limitation of our study is the lack of data on functional outcomes such as frailty, independence, or nutritional status, which could further characterize the benefits and risks of kidney transplantation in older recipients. Lastly, the cut-off age of 60 years was used to distinguish between older and younger patients, while many studies have used the age of 65 years or even higher. Our choice was made based on the United Nations definition, which in our opinion is a reliable source. Moreover, expected remaining years of life for KT recipients aged >60 years are similar to those of people older than 75 years old in the general population [2].

## 5. Conclusions

Surgical complications, with emphasis on urological problems, DGF, and infectious complications are more common in patients aged ≥60 years. Thus, a careful approach should be applied in this group. PTDM and cardiac events are also more frequently observed in senior patients. No significant differences in terms of CMV replication, BKV replication, and BPAR were detected. The estimated glomerular filtration rate was lower in the older group, although it remained an acceptable outcome. One-year patient and graft survival were comparable between both groups, which indicates that kidney transplantation in older recipients is a relatively safe procedure.

## Figures and Tables

**Table 1:** Comparison of the baseline characteristics between older and younger recipients.

Variable	Patients <60 years	Patients ≥60 years	MD/RR (95% CI)	*p*
N	195 (100.0)	75 (100.0)	-	-
Age, years	41.21 ± 10.14	64.99 ± 3.62	23.78 (22.13;25.43)	<0.001
Sex, female	80 (41.0)	29 (38.7)	0.94 (0.68;1.31)	0.829
ESKD cause				<0.001
DM	18 (9.2)	13 (17.3)		
ADPKD	26 (13.3)	12 (16.0)		
HT	8 (4.1)	6 (8.0)		
Chronic glomerulonephritis	89 (45.6)	17 (22.7)		
Undetermined	15 (7.7)	19 (25.3)		
Others	24 (12.3)	5 (6.7)		
Urological causes	15 (7.7)	3 (4.0)		
BMI, kg/m^2^	24.80 ± 4.35	26.57 ± 4.00	1.77 (0.63;2.91)	0.002
Dialysis				0.249
HD	135 (69.2)	61 (81.3)		
PD	19 (9.7)	4 (5.3)		
HD + PD	14 (7.2)	4 (5.3)		
Preemptive	27 (13.8)	6 (8.0)		
Dialysis duration (HD + PD), months	24.50 (13.00;43.00)	33.00 (16.00;49.00)	8.50 (−1.00;10.00)	0.076
PRA	1 (0.5)	0 (0.0)	-	0.251
mean PRA (%)	1,84 ± 8,24	3,64 ± 15,58	-	-
HLA mismatches	2.93 ± 1.24	3.25 ± 1.23	0.33 (−0.01;0.66)	0.054
Hypertension (nr of medicines)	2.50 ± 1.57	2.72 ± 1.13	0.22 (−0.12;0.56)	0.198
Cardiovascular diseases (prior to transplantation)	14 (7.2)	28 (37.3)	5.20 (2.90;9.32)	<0.001
Diabetes mellitus (prior to transplantation)	23 (11.8)	26 (34.7)	2.94 (1.79;4.82)	<0.001
Induction immunosuppression				0.014
Thymoglobulin induction	53 (27.2)	8 (10.7)		
Basiliximab induction	21 (10.8)	9 (12.0)		
Donor type				<0.001
DDKT	141 (72.3)	71 (94.7)		
LDKT	54 (27.7)	4 (5.3)		
DDKT expanded criteria ^1^	19 (13.5)	39 (54.9)	4.08 (2.55;6.51)	<0.001
Donor age, years	45.16 ± 12.81	57.53 ± 12.82	12.37 (8.94;15.80)	<0.001
Diabetes mellitus (donor)	6 (3.1)	4 (5.3)	1.73 (0.50;5.97)	0.472
Hypertension (donor)	50 (25.6)	34 (45.3)	1.77 (1.25;2.50)	0.003
Arterial vasculopathy (donor)	25 (12.8)	21 (28.0)	2.18 (1.30;3.66)	0.005

ADPKD—autosomal dominant polycystic kidney disease, BMI—body mass index, CI—confidence interval, DDKT—deceased donor kidney transplant, ESKD—end-stage kidney disease, HD—hemodialysis, HLA—human leukocyte antigen, HT—hypertension, LDKT—living donor kidney transplant, MD—mean or median difference (≥60 years vs. <60 years), PD—peritoneal dialysis, PRA—panel reactive antibody, RR—relative risk (≥60 years vs. <60 years). Data presented as mean ± SD or median (IQR) in case of numeric variables, depending on distribution and n (%) in case of categorical variables. Groups compared with t-Student test, t-Welch test, Mann-Whitney U test, Pearson’s chi-square test or Fisher’s exact test, as appropriate. Values in parentheses represent either percentages (for categorical variables) or 95% confidence intervals (for mean/median differences or relative risks), as appropriate. ^1^ Proportion calculated to DDKD patients.

**Table 2:** Comparison of post-transplantation outcomes and complications between groups.

Variable	Patients <60 years	Patients ≥60 years	MD/RR (95% CI)	*p*
No of hospitalizations within 12 months	1.00 (0.00;2.00)	2.00 (1.00;3.00)	1.00 (0.00;1.00)	0.002
Proportion of patients hospitalized due to infection within 12 months	41 (21.0)	26 (34.7)	1.65 (1.09;2.49)	0.030
Bacterial	32 (16.4)	20 (26.7)	1.62 (0.99;2.66)	0.082
Viral	6 (3.1)	4 (5.3)	1.73 (0.50;5.97)	0.472
Not identified	8 (4.1)	9 (12.0)	2.92 (1.17;7.30)	0.035
Fungal	3 (1.5)	1 (1.3)	0.87 (0.09;8.20)	>0.999
Infection-related hospitalizations (per patient/year) *	0.00 (0.00;0.00)	0.00 (0.00;1.00)	0.00 (0.00;0.00)	0.019
Surgical complication	33 (16.9)	23 (30.7)	1.81 (1.14;2.87)	0.020
Vascular	13 (6.7)	11 (14.7)	2.20 (1.03;4.69)	0.067
Urological	15 (7.7)	10 (13.3)	1.73 (0.82;3.69)	0.231
Wound	9 (4.6)	4 (5.3)	1.16 (0.37;3.64)	0.759
Graft function				
Immediate Graft Function (IGF)	128 (65.6)	29 (38.7)	-	<0.001
Delayed graft function (DGF)	65 (33.3)	43 (57.3)		
Primary non-function (PNF)	2 (1.0)	3 (4.0)		
DGF length, days	4.00 (2.00;7.00)	4.00 (3.00;6.50)	0.00 (−1.00;1.00)	0.693
BPAR	43 (22.1)	15 (20.0)	0.91 (0.54;1.53)	0.840
PTDM	40 (20.5)	22 (29.3)	-	<0.001
no DM after Tx	132 (67.7)	28 (37.3)		
Cardiac events	16 (8.2)	14 (18.7)	2.28 (1.17;4.43)	0.025
Arrhythmia	10 (5.1)	8 (10.7)	2.08 (0.85;5.07)	0.173
MI	3 (1.5)	3 (4.0)	2.60 (0.54;12.60)	0.353
New onset HF	4 (2.1)	3 (4.0)	1.95 (0.45;8.51)	0.401
CMV replication	36 (18.5)	20 (26.7)	1.44 (0.90;2.33)	0.186
BKV replication	27 (13.8)	13 (17.3)	1.25 (0.68;2.29)	0.595
Remuzzi score [0–12]	1.00 (0.00;2.00)	2.00 (1.00;3.00)	1.00 (0.00;1.00)	<0.001
Remuzzi score (mean)	1.47	2.53	-	-
sCr at 12 months follow-up	1.40 (1.10;1.77)	1.55 (1.10;2.00)	0.15 (−0.04;0.25)	0.165
eGFR at 12 months follow-up	56.81 ± 20.48	45.02 ± 19.36	−11.79 (−17.32;−6.25)	<0.001
Patient’s death within 12 months	3 (1.5)	2 (2.7)	1.73 (0.30;10.17)	0.620
Patient’s 12 months survival	192 (98.5)	73 (97.3)	0.99 (0.95;1.03)	0.620
Graft 12 months survival	189 (96.9)	71 (94.7)	0.98 (0.92;1.04)	0.472
Characteristics regarding initial hospitalization				
Length of initial hospitalization, days	14.00 (10.00;21.00)	19.00 (13.00;27.00)	5.00 (2.00;6.00)	<0.001
sCr at discharge	1.61 (1.29;2.15)	1.68 (1.30;2.20)	0.06 (−0.10;0.21)	0.520
eGFR at discharge	46.00 (33.00;62.25)	39.50 (25.50;56.25)	−6.50 (−13.00;−3.00)	0.004
Infection during 1st hospitalization	60 (30.8)	33 (44.0)	1.43 (1.03;1.99)	0.057
Urinary tract infection	41 (21.0)	26 (34.7)	1.65 (1.09;2.49)	0.030
Wound infection (during	1 (0.5)	2 (2.7)	5.20 (0.48;56.50)	0.188
Other	19 (9.7)	6 (8.0)	0.82 (0.34;1.98)	0.835

BKV—BK polyomavirus, BPAR—biopsy-proven acute rejection, CI—confidence interval, CMV—cytomegalovirus, DGF—delayed graft function, DM—diabetes mellitus, eGFR—estimated glomerular filtration rate, IGF—immediate graft function, MD—mean or median difference (≥60 years vs. <60 years), No—number, PNF—primary non-function, PTDM—post-transplant diabetes mellitus, RR—relative risk (≥60 years vs. <60 years), sCr—serum creatinine, Tx—transplantation. Data presented as mean ± SD or median (IQR) in case of numeric variables, depending on distribution and n (%) in case of categorical variables. Groups compared with t-Student test, Mann-Whitney U test, Pearson’s chi-square test or Fisher’s exact test, as appropriate. * Infection-related hospitalizations are shown as mean events per patient/year. Values reported as “0.00” indicate mean values <0.01 after rounding.

## Data Availability

The data presented in this study are available on request from the corresponding author due to privacy reasons.

## Abbreviations

The following abbreviations are used in this manuscript:

ADPKD	autosomal dominant polycystic kidney disease
ATG	thymoglobulin
BPAR	biopsy-proven acute rejection
BMI	body mass index
BKV	polyomavirus BK
CNI	calcineurin inhibitors
CMV	cytomegalovirus
CVD	cardiovascular diseases
DGF	delayed graft function
DDKT	deceased donor kidney transplant
DM	diabetes mellitus
eGFR	estimated glomerular filtration rate
ESKD	end-stage kidney disease
HD	hemodialysis
HLA	human leukocyte antigen
HT	hypertension
IGF	immediate graft function
IQR	interquartile range
KT	kidney transplants
LDKT	living donor kidney transplant
MD	mean/median difference
No	number
PRA	panel reactive antibody
PNF	primary non-function
PTDM	post-transplant diabetes mellitus
RR	relative risk
SD	standard deviation
sCr	serum creatinine
Tx	transplantation
